# Special issue: “Metamaterials and Plasmonics in Asia”

**DOI:** 10.1515/nanoph-2025-0162

**Published:** 2025-04-18

**Authors:** Shumin Xiao, Lei Zhou, Bumki Min, Takuo Tanaka, Atsushi Sanada

**Affiliations:** 47822 Harbin Institute of Industry, HIT Campus, University Town, Nanshan District, Harbin 150001, China; Harbin Institute of Technology, HIT Campus, University Town, Nanshan District, Harbin 150001, China; Surface Physics Laboratory and Physics Department, Fudan University, Shanghai 200433, China; Department of Mechanical Engineering, Korea Advanced Institute of Science and Technology (KAIST), Daejeon, The Republic of Korea; RIKEN – Metamaterials Lab., 2-1 Hirosawa, Wako, Saitama 351-0198, Japan; Osaka University, Toyonaka, Osaka, Japan

This special issue, titled “Metamaterials and Plasmonics in Asia”, is originated from the eighth A3 Metamaterials Forum, which took place in Shenzhen, China, from July 23 to 25, 2024. The key aim of this special issue is to celebrate the remarkable contributions of Asian researchers to these transformative fields and highlights the region’s growing influence in shaping the future of photonics and nanotechnology. The A3 Metamaterials Forum is an annual gathering that brings together prominent researchers from three Asian nations – Korea, Japan, and China – who specialize in the field of metamaterials. The forum covers a wide range of topics, including the development and application of metamaterials and metasurfaces in electromagnetics, acoustics, optical and other related systems.

As we gather in Shenzhen for the A3 Metamaterials Forum, we are reminded of the power of collaboration and the importance of fostering international partnerships. The rapid progress in metamaterials and plasmonics is a testament to the collective efforts of the global scientific community, and Asia’s contributions have been pivotal in driving this progress. This special issue not only highlights the achievements of Asian researchers but also serves as a platform for inspiring future innovations and collaborations.

Metamaterials, with their engineered properties that defy natural limitations, and plasmonics, which harnesses the unique interactions between light and free electrons in metals, have opened new frontiers in science and technology. From superlens that breaks the diffraction limit to invisibility cloaks and ultra-compact photonic circuits, these fields have revolutionized our abilities to control light at the nanoscale. Recently, metasurfaces, planar version of metamaterials, has significantly expanded the boundaries of what is possible in optical metamaterials, plasmonic devices, and their applications in sensing, imaging, energy, and communications.

This special issue brings together a collection of cutting-edge research articles, reviews, and perspectives from leading scientists across Asia and beyond. It showcases the diversity and depth of ongoing work in metamaterials, plasmonics and metasurfaces, ranging from fundamental theoretical studies to innovative experimental demonstrations and real-world applications. Topics include but are not limited to:

Three review articles are presented in this special issue. Huang et al. provide an overview of recent progresses in all-optical analog differential operation and provide outlook on the challenges and prospects in information processing empowered by meta-devices [1]. Li et al. summarize metasurface-enhanced biomedical spectroscopy [2]. Choi et al. discuss the basic theory, experiments, and applications for topological guided-mode resonances [3].

In addition to these review articles, this special issue also contains 23 research papers. Guan et al. proposed a technology for ultrasensitive circular dichroism spectroscopy based on coupled quasi-bound states in the continuum [4]. Shin et al. realized data-efficient prediction of OLED optical properties by transfer learning method [5]. Zhou et al. developed a semimetal-dielectric-metal metasurface for infrared camouflage with high-performance energy [6]. Park et al. reported deep-subwavelength engineering of stealthy hyperuniformity [7]. Zhang et al. generated tunable structural colors based on grayscale lithography and conformal coating of phase change materials [8]. Liu et al. proposed a general recipe to observe non-abelian gauge field in metamaterials [9]. Chen et al. demonstrated a free-form catenary-inspired meta-couplers for ultra-high or broadband vertical coupling [10]. Tomoshige et al. discussed the enhancement of photoluminescence for strongly coupled single molecule-plasmonic nanocavity [11]. Jo et al. developed a spectral Hadamard microscopy with metasurface-based patterned illumination [12]. Lee and Kang reported tunneling of two-dimensional surface polaritons through plasmonic nanoplates on an atomically thin platform [13]. Jeong et al. also reported a highly sensitive microdisk laser sensor for refractive index sensing via periodic meta-hole patterning [14]. Qi et al. proposed a scaled transverse translation by planar optical elements for sub-pixel sampling and remote super-resolution imaging [15]. Li et al. demonstrated a hyperbolic polariton-coupled emission optical microscopy [16]. Zhu et al. reported a broadband perfect Littrow diffraction metasurface under large-angle incidence [17]. Kang et al. discussed the role of complex energy and momentum in open cavity resonances [18]. Kim et al. discussed the effect of intermediate mirrors in enhancing the efficiency of perovskite tandem solar cells [19]. Cheng et al. introduced a tunable metadevice for Large Depth of Field Quantitative Phase Imaging [20]. Lee et al. demonstrated an enhanced terahertz magneto-plasmonic effect enabled by epsilon-near-zero iron slot antennas [21]. Shen et al. proposed baseline-free structured light 3D imaging using a metasurface double-helix dot projector [22]. Kim et al. developed leveraging large language models for the design of nanophotonic devices [23]. Dai et al. proposed a generic scheme to realize bi-functional meta-holography with high efficiencies [24]. Jang et al. proposed a dielectric metasurfaces based on a phase singularity in the region of high reflectance [25]. Hasanli et al. reported exceptional points in a passive strip waveguide [26].

We extend our gratitude to the authors, reviewers, and editorial team whose dedication and expertise have made this special issue possible. We also thank the A3 Metamaterials Forum organizers for providing a forum to discuss and celebrate these exciting developments. As we look to the future, we are confident that the insights and breakthroughs presented here will pave the way for new discoveries and applications, further solidifying Asia’s role as a global leader in metamaterials and plasmonics.

Let this special issue be a testament to the ingenuity and creativity of researchers in Asia and beyond, and a catalyst for even greater achievements in the years to come.
